# Milk protein for improved metabolic health: a review of the evidence

**DOI:** 10.1186/1743-7075-10-46

**Published:** 2013-07-03

**Authors:** Robin A McGregor, Sally D Poppitt

**Affiliations:** 1 School of Biological Sciences, University of Auckland, Auckland 1010, New Zealand; 2 University of Auckland Human Nutrition Unit, 18 Carrick Place, Mt Eden, Auckland 1024, New Zealand; 3 Department of Medicine, University of Auckland, Auckland 1010, New Zealand; 4 Riddet Institute, Palmerston North 4442, New Zealand

**Keywords:** Dairy protein, Milk, Whey protein, Metabolic health, Hyperglycaemia, Dyslipidaemia, Blood pressure, Inflammation, Body weight

## Abstract

Epidemiological evidence shows that consumption of dairy products is associated with decreased prevalence of metabolic related disorders, whilst evidence from experimental studies points towards dairy protein as a dietary component which may aid prevention of type 2 diabetes (T2DM). Poor metabolic health is a common characteristic of overweight, obesity and aging, and is the forerunner of T2DM and cardiovascular disease (CVD), and an ever increasing global health issue. Progressive loss of metabolic control is evident from a blunting of carbohydrate, fat and protein metabolism, which is commonly manifested through decreased insulin sensitivity, inadequate glucose and lipid control, accompanied by a pro-inflammatory environment and hypertension. Adverse physiological changes such as excess visceral adipose tissue deposition and expansion, lipid overspill and infiltration into liver, muscle and other organs, and sarcopaenia or degenerative loss of skeletal muscle mass and function all underpin this adverse profile. ‘Sarcobesity’ and sarcopaenic diabetes are rapidly growing health issues. As well as through direct mechanisms, dairy protein may indirectly improve metabolic health by aiding loss of body weight and fat mass through enhanced satiety, whilst promoting skeletal muscle growth and function through anabolic effects of dairy protein-derived branch chain amino acids (BCAAs). BCAAs enhance muscle protein synthesis, lean body mass and skeletal muscle metabolic function. The composition and processing of dairy protein has an impact on digestion, absorption, BCAA kinetics and function, hence the optimisation of dairy protein composition through selection and combination of specific protein components in milk may provide a way to maximize benefits for metabolic health.

## Introduction

Poor metabolic health represents an ever increasing global epidemic based on estimates from countries as far ranging as the US and China [1,2]. Encompassed within metabolic health are the cluster of interrelated adverse metabolic markers of hyperglycaemia, dyslipidaemia and hypertension which alongside central or abdominal obesity have been termed the metabolic syndrome [3,4]. Individuals with metabolic syndrome are at twice the risk of developing cardiovascular disease (CVD) within 5-10 years, in addition to a 5-fold increase in risk of developing T2DM [3], and therefore maintenance of good metabolic health is critically important.

A continuum of metabolic health exists from young, lean, healthy individuals with good physiological control to those with impaired metabolic regulation who are commonly overweight or obese, as well as older. Progressive loss of metabolic control is characterized by a range of physiological changes which include excess adipose deposition, lipid overspill, infiltration and accumulation in key organs such as liver and skeletal muscle, alongside blunting of carbohydrate (CHO), fat and protein metabolism, decreased insulin sensitivity and hyperglycaemia, dyslipidaemia, increased inflammation, impaired endothelial function [5], and blunted muscle protein synthesis and decreased muscle mass, structure and function [6]. Multiple factors may contribute to the progressive loss of metabolic control, but obesity, ageing and physical inactivity are recognized as major drivers of these changes in metabolic health [3,4].

Obesity is of particular importance and a prolonged positive energy balance with subsequent lipid deposition and expansion of adipose depots [7], particularly visceral depots which secrete inflammatory cytokines, play a role in insulin resistance and decreased insulin-mediated glucose uptake [8]. Lipid turnover is decreased and mitochondrial oxidation is suppressed in obese subjects, promoting intracellular accumulation of lipids and the buildup of deleterious lipid metabolites in multiple tissues including skeletal muscle, liver, pancreatic beta cells, kidney and hypothalamus amongst others [9]. Subsequently, infiltration of inflammatory cells to clear toxic metabolic byproducts is accompanied by release of inflammatory cytokines that inhibit metabolic signaling pathways [10], as well as promote cell death, tissue fibrosis and functional impairment. The recommended treatment to improve metabolic health includes changes in diet and physical activity, which promote adipose tissue loss, enhance metabolically active skeletal muscle mass and hence improve metabolic control [5]. Energy restricted diets are widely recommended for weight-loss in overweight or obese individuals with poor metabolic health, however ~25% of body weight loss can be attributable to decreases in skeletal muscle mass [11-13]. Loss of skeletal muscle is undesirable because it is essential for mobility and activities of daily living. In addition, skeletal muscle also plays a major role in glycaemic control accounting for up to 75% of tissue glucose uptake. Mitochondrial oxidative capacity is decreased in obese skeletal muscle, as well as in T2DM, possibly due to underlying physical inactivity [14].

Advancing age and a sedentary lifestyle are also risk factors for a gradual loss of skeletal muscle mass, function and in turn muscle strength. The problem is commonly compounded by increased adipose tissue accumulation and myocellular lipid infiltration which provides the basis of sarcopenic (accelerated muscle loss) obesity, and which in turn may drive insulin resistance and increase metabolic risk [15]. Intramyocellular lipid accumulation in obese individuals appears to be difficult to reverse through weight-loss interventions [16]. Activation of skeletal muscle protein anabolism appears to be blunted in both the obese [17] and the elderly, although again this may be attributable to a common underlying physical inactivity and insulin resistance [6,18]. If metabolic health does not improve with diet or exercise interventions, pharmacological agents can be resorted to in order to manage dyslipidaemia, hypertension and hyperglycemia and loss of metabolic homeostasis.

Currently, there is considerable interest in the use of dairy proteins as supplements or in conjunction with lifestyle changes to improve metabolic health [19-23]. Evidence from some epidemiological studies suggest that greater consumption of dairy products is associated with lower risk of metabolic related disorders and CVD [20,24]. Multiple dairy components in milk such as whey protein, casein and minerals have been posited as drivers of these beneficial effects [20], and there is a growing body of intervention studies assessing the effects of cow’s milk-derived proteins [23] or peptides on metabolic health [25]. The focus of many of these interventions has been the whey component of milk, which may act to improve cardiometabolic risk factors. Whey protein has been shown to be an insulin secretagogue [26], as well as to improve body weight and adiposity through increased satiety [19]. In addition to a dietary strategy to promote adipose loss, dairy proteins have also been shown to increase skeletal muscle mass through stimulation of muscle protein synthesis [27]. Peptides derived from dairy protein have also been widely investigated as potential inhibitors of angiotensin-converting enzyme (ACE), thereby regulating blood pressure [28], and may influence activation of the innate immune system and inflammation [29].

### Milk processing, protein composition and kinetics

Dairy protein consumed by humans is predominantly from cow’s milk, which consists of around 80% (w/w) casein, 20% (w/w) whey proteins and is also a rich source of minerals such as calcium. The casein in cow’s milk comprises alpha-s1, alpha-s2, beta and kappa-casein, whilst whey comprises multiple globular proteins including beta-lactoglobulin, alpha-lactalbumin, lactoferrin, immunoglobulins, serum albumin, glycomacropeptide, enzymes and growth factors. All of these components have potential to contribute to the observed association between increased consumption of dairy products and decreased risk of metabolic disease observed in several epidemiological studies [20,30,31].

#### Cow’s milk processing

Cow’s milk processing is an important factor determining the composition, concentration and physiological effects of whey protein or casein [32,33]. Milk is commonly separated into different protein fractions for different food applications [34]. Milk protein concentrate (MPC), produced by ultrafiltration of skimmed milk, contains both casein and whey proteins in similar proportions to whole milk, but the total amount of protein, lactose and mineral content may vary between different MPC formulations. Micellar casein can be extracted from milk protein concentrate by further ultrafiltration. Casein is produced from skim milk by acid precipitation or enzymatic coagulation, washing and drying. Caseinates are produced by treatment of acidified or coagulated casein curd with alkali such as sodium hydroxide or calcium hydroxide, which forms sodium or calcium caseinates respectively; caseinates contain ~90% protein. Whey protein concentrate is produced by coagulation of milk with the enzyme rennet or acid, resulting in separation of curds and whey, further ultrafiltration and drying produces whey protein concentrates containing ~25-80% protein. Additional processing can produce whey protein isolates containing >90% protein with very low amounts of lactose and lipids. Hydrolysis with enzymes or acids provides a way to breakdown the structure of whey or casein. In metabolic-related studies a range of processed milk proteins have been used including milk protein concentrate, micellar casein, casein, sodium caseinate, calcium caseinate, casein hydrolysate, whey protein concentrate, whey protein isolate and whey protein hydrolysate, as well as a range of whey and casein peptides.

#### Amino acid profile of milk proteins

Whey protein and casein are both classified as high quality proteins based on human amino acid (AA) requirements, digestibility and their bioavailability. They contain a relatively high proportion of indispensible AAs, score higher than most other protein sources across a wide range of assessment methods including the protein digestibility corrected AA score (PDCAAS) [35] and recently developed digestible indispensable amino acid score (DIAAS) method [36]. Nevertheless, differences in the physiological effects of whey protein and casein have been attributed to differences in their AA composition [37]. Whey protein contains a higher proportion of the branched chain amino acids (BCAA) leucine, isoleucine and valine compared to casein [38]. The BCAAs alone and in particular leucine have been shown to trigger a potent increase in protein synthesis in T2DM [39]. Among the other indispensible or essential amino acids (EAAs), casein contains a higher proportion of histidine, methionine, phenylalanine and valine than whey protein [38]. In addition, casein also contains a higher proportion of several non-EAAs including arginine, glutamic acid, proline, serine and tyrosine [38].

#### Gastric emptying, absorption and serum kinetics of milk proteins

Whey protein is reported to be absorbed faster than casein [40]. The lower absorption rate of casein in its native micellar form is because the low pH conditions in the stomach cause casein to clot and delays gastric emptying [41]. Therefore, plasma AAs are more rapidly elevated following whey protein consumption, whereas changes in plasma AAs are lower and more sustained following micellar casein consumption [40]. Processing of whey protein or casein fractions via hydrolysis can markedly influence absorption and subsequent plasma AA profiles. A casein hydrolysate is reported to be absorbed more rapidly than intact micellar casein, resulting in a greater increase in plasma AAs [42]. Conversely, whey protein hydrolysate intake is reported to result in similar plasma AA levels compared to whey protein concentrate [43], because of similar rapid rates of gastric emptying and absorption. The processing of micellar casein by acidification and then neutralization with alkali such as sodium hydroxide or calcium hydroxide to form caseinates also markedly alters plasma AA profiles compared to micellar casein [44].

### Milk proteins, insulin secretion and glucose control

#### Insulinotropic effects

Insulin is sensitive to both the composition and concentration of plasma AAs, hence both whey and casein ingestion stimulate increased insulin secretion [45,46]. Ingestion of whey protein leads to more rapid secretion of insulin than micellar casein [40], however, hydrolysis of casein speeds up the absorption of AAs and secretion of insulin relative to the micellar form of casein [42]. Insulin has wide-ranging direct and indirect effects on CHO, fat and protein metabolism, including stimulation of glucose uptake, glycogen synthesis, lipid uptake, triglyceride (TG) synthesis, protein synthesis and inhibition of protein breakdown, lipolysis and gluconeogenesis. Therefore, the stimulation of insulin secretion by different milk proteins may make a significant contribution to metabolic effects in insulin sensitive tissues and in particular skeletal muscle anabolism. Prolonged, elevated fasting glucose is a key metabolic risk factor, one of the main characteristics of T2DM and associated with an increased risk of CVD [47]. Hyperglycaemia develops with increased insulin resistance and failure of insulin secretion.

#### Postprandial glycaemia

Management of non-fasting or postprandial glucose response is important to minimize prolonged exposure to high blood glucose levels in individuals both with and without T2DM [48]. Plasma glucose can increase protein glycosylation, nonenzymatic glycation products and the generation of free radicals, as well as decrease nitric oxide production leading to damage to the macro- and microvasculature [48]. Of significant importance to dairy are the milk proteins whey and casein which stimulate insulin release, and have the potential to alter tissue glucose uptake and suppress postprandial blood glucose excursions [43,45,46,49-51].

Many of the studies on the insulinotropic inducing properties of whey protein or casein have been conducted in healthy men rather than individuals with impaired glucose control [43,45,46,49-51]. The AA profile of whey protein is suggested to contribute to its insulinotropic effects, however, the protein format has been shown to have variable effects on insulin secretion. AA-supplemented drinks containing several insulinotropic AAs found at high levels in whey protein (e.g. leucine, isoleucine, valine, lysine, threonine) are reported to result in similar insulinaemic and glycaemic responses [46]. A recent study showed co-ingestion of these AAs with 9 g whey protein however did not further augment the suppression of postprandial glucose after a CHO meal [52], which may be due to a ceiling in the stimulation of insulin by free AAs and AAs derived from intact protein. One study has shown that insulinotropic effects between intact whey protein and whey protein hydrolysate does not vary in healthy individuals at doses of 20-50 g protein [43]. Conversely, in a different study whey protein concentrate but not hydrolysate decreased postprandial blood glucose and insulin responses in a dose dependent manner (10-40 g) following an *ad libitum* mixed meal, although the authors noted that this was most likely because food intake was decreased at this free choice meal [49], hence confounding these effects.

In individuals with T2DM, 18 g whey protein added to breakfast or lunch resulted in greater insulinotropic responses, circulating levels of the gut peptide glucose-dependent insulinotropic polypeptide (GIP), and suppression of postprandial glycaemia than following an isoenergetic non-dairy protein (lean ham and lactose) [53]. 55 g whey protein ingested before or with a CHO lunch also suppressed postprandial glucose in T2DM patients [54], triggering greater insulinotropic, and gut peptide (GIP and cholecystokinin, CCK) responses [54]. Gastric emptying was only inhibited with pre-meal whey protein ingestion, although there was no evidence that this was any more effective for postprandial glycaemic control than ingestion with a meal [54]. It is of considerable interest that the acute effects of whey protein on postprandial blood glucose are comparable to sulfonylureas and other insulin secretagogues used for the pharmaceutical management of hyperglycaemia in T2DM. Sulfonylureas stimulate increased secretion of (pro) insulin by binding to ATP-dependent potassium channels in pancreatic β-cells [55]. Therefore, there is a rationale for regular whey protein ingestion before or with meals to manage postprandial glycaemic responses in individuals with poor metabolic control or T2DM.

Effects of casein may be less consistent. In an overweight group with T2DM, consumption of a casein hydrolysate (~30 g) and leucine (~10 g) beverage after breakfast, lunch and dinner decreased prevalence of hyperglycaemia over the course of 24 hours [56]. Yet in another study of patients with long-standing T2DM, higher 40 g dose casein hydrolysate given at each main meal did not improve the prevalence of hyperglycemia over 24 hours [57], possibly attributable to β-cell impairment characteristic of long-term T2DM. Although, even in long-standing T2DM however, there is some evidence that the insulin secretory mechanism is retained and can be re-activated by ingestion of free AA and protein mixtures including free leucine, free phenylalanine and wheat protein hydrolysate [58]. Possibly whey protein or casein could improve hyperglycemia over 24 hours in individuals with metabolic syndrome or early T2DM characterized by insulin resistance but still functional β-cells.

#### Chronic fasting glycaemia

To date there have been few randomized controlled trials of longer-term whey protein or casein supplementation on glycaemic control. In the only study of which we are aware in overweight and obese individuals, 12 weeks of 54 g/day whey protein isolate or sodium caseinate supplementation without any lifestyle intervention resulted in a decrease in fasting blood insulin levels and insulin resistance, but not fasting blood glucose levels [59], compared to 12 weeks of glucose supplementation. Most individuals at the start of the trial had borderline impaired glucose tolerance (IGT) as well as other metabolic risk factors including high TG, low HDL-C and high waist circumference [59]. Further long-term trials of whey protein or casein in individuals with IGT are warranted based on the evidence from acute trials showing milk proteins suppress postprandial glycaemia chronic fasting hyperinsulinaemia and insulin resistance.

### Milk proteins and blood lipids

#### Postprandial dyslipidaemia

Dyslipidaemia is one of the main metabolic risk factors associated with CVD risk [60]. Metabolic syndrome is mainly characterized by elevated levels of TG and low levels of HDL-C [4]. Even in the absence of overt dyslipidaemia in metabolic syndrome, postprandial lipaemia can cause transient impairment in endothelial function and other adverse outcomes [61]. Dairy proteins have potential to suppress postprandial lipaemia due to their insulinotropic effects, as insulin is known to inhibit hormone-sensitive lipase and release of FFA [62]. However, studies on the effects of milk protein ingestion on postprandial lipaemia and chronic dyslipidaemia have produced mixed findings [63-70]. Two studies in healthy normolipaemic young men and women reported that 50 g sodium caseinate suppressed the postprandial response following a 70 g fat bolus, decreasing diet-derived chylomicrons and FFAs independent of gastric emptying, compared to an oligosaccharide bolus [64,65]. In another study of normolipaemic young men and women, however, ingestion of a lower dose of 23 g casein did not significantly modulate the postprandial lipaemic response to a 40 g fat meal [63].

In a study of obese post-menopausal women, 45 g whey protein isolate or sodium caseinate with breakfast both decreased the postprandial appearance of chylomicron-derived TG as well as the TG:ApoB48 ratio [66]. There was no change in total cholesterol, LDL-C, HDL-C, FFA or ApoB48 alone. Since postprandial effects appear to predominate, it was unsurprising that the cholesterol moieties did not change postprandially. When investigating the effect of different types of dairy proteins, also in obese individuals, a similar bolus of 45 g whey protein isolate, whey protein hydrolysate, alpha-lactalbumin or casein glycomacropeptide (GMP) did not differentially alter postprandial lipaemia following a fatty meal, although, whey hydrolysate marginally decreased postprandial suppression of FFAs relative to the other dairy fractions [68]. In T2DM patients, 45 g whey protein has been shown to suppress postprandial TG, FFA and rate of appearance of chylomicron-rich lipoproteins after a high-fat meal compared with casein and also non-dairy (cod and gluten) proteins [69], whilst a second study in T2DM patients found no response to 45 g casein on the postprandial TG response following a similar high-fat meal [67].

As yet the mechanism underpinning the effect of dairy protein on postprandial lipaemia is not well understood. Nevertheless, the evidence of acute effects of dairy protein, particularly whey, on postprandial lipaemia provides a rationale for longer-term supplementation trials to manage dyslipidaemia.

#### Chronic fasting dyslipidaemia

There have to date been few long-term randomized trials investigating fasting lipids in healthy individuals or those with adverse metabolic risk. In a study of metabolic syndrome, a significant decrease in fasting TG was demonstrated following a 3 month period of supplementation of 15 g/day of a fermented whey product, whey malleable protein matrix (MPM, comprising whey proteins, peptides, a probiotic, polysaccharides and calcium) [70], as were other risk factors but only in individuals with high pre-existing metabolic risk. Metabolic outcomes in this study may have been driven by weight loss since body weight also decreased following MPM supplementation. In addition, dairy protein supplementation may provide an indirect way to improve blood lipid profiles when used in conjunction with low fat or low energy diets to induce weight-loss. Certainly there is experimental evidence that whey protein can decrease lipid infiltration into the liver, for example in rodent models of non-alcoholic fatty liver disease (NAFLD) [71], where hepatic TG is normalized through dietary treatment. Dairy protein products appear to have potential to decrease dyslipidaemia in high-risk individuals, but more studies are needed.

### Milk proteins, vascular reactivity and blood pressure

In epidemiological studies, consumption of dairy products including milk and yogurt is associated with decreased risk of hypertension, purported to be driven by a high content of bioactive peptides [20,72]. Whey protein and casein both contain the bioactive peptides lactokinins or caseinkinins respectively. *In-vitro* experiments indicate these peptides can inhibit ACE activity [25]. ACE is a rate limiting enzyme in the conversion of angiotensin I to angiotensin II responsible for vasoconstriction. Therefore, lactokinins or caseinkinins both have potential to lower blood pressure. Vascular reactivity is important for glucose disposal and regulation of blood flow, but is impaired in patients with metabolic syndrome due to decreased insulin-stimulated vasodilation via the eNOS pathway and suppressed NO production in the vascular endothelium [73]. In a recent study of older overweight adults with impaired vascular endothelial function, 5 g of a novel whey protein-derived extract increased brachial artery flow-mediated dilation over a 2 hour period compared to a placebo [74], although notably without inhibition of ACE activity or alteration of circulating endothelium factors including plasma NO, prostacyclin metabolites or endothelin-1 [74]. Evidence for improvements in blood pressure has been steadily growing in the majority [75-80], although not all trials [81]. In a study of overweight postmenopausal women, 45 g whey protein isolate and sodium caseinate had no effect on postprandial arterial stiffness or on blood pressure over 6 hours [81]. Conversely, in overweight men and women with mild hypertension, 6 weeks of supplementation with 28 g/day whey protein concentrate or hydrolysate decreased both systolic and diastolic pressure as well as mean arterial pressure [75]. Whilst in a group of pre- and hypertensive individuals, 6 weeks of supplementation with 20 g/day whey protein hydrolysate was shown to further decrease blood pressure compared to intact whey protein [76], despite no detectable differences in ACE activity. Interestingly, in a longer 12 week trial of overweight, normo- and hypertensive men and women, 54 g/day whey protein isolate or sodium caseinate were both reported to decrease blood pressure and arterial stiffness compared to a glucose control [77]. Reductions in systolic blood pressure have also been found following supplementation with fermented or immune modified milks [78,79], and clinical trials of whey peptides on blood pressure and vascular function have produced some promising findings [80].

### Milk proteins, immune response and inflammation

A chronic low-grade inflammatory state accompanies obesity, evident from both elevated systemic inflammatory markers in serum and infiltration of inflammatory cells into tissues. In obese humans, serum levels of pro-inflammatory cytokines are elevated and peripheral blood mononuclear cells are activated [82]. Inflammatory cytokines can increase insulin resistance and inhibit glucose uptake in peripheral tissues, as well as increase proteolysis of skeletal muscle cells. Evidence from *in-vitro* studies suggests that dairy proteins and milk derived peptides have immunosuppressive or immunostimulatory effects [29]. Kappa-casein has been reported to suppress lymphocyte proliferation induced by T and B cell mitogens [83]. Whey protein and its hydrolysates have been to inhibit the proliferation of lymphocytes, without the induction of apoptosis [84]. When mitogens or antigens activate T lymphocytes they produce cytokines and upregulate cell surface immune receptors. Whey proteins lactoferrin and lactoperoxidase have been shown to suppress interferon secretion from mitogen activated lymphocytes [85]. Animal studies indicate whey protein and casein or their peptides have immunomodulatory effects *in-vivo,* but these are not always in concordance with effects observed *in-vitro*[29]. There have been very few studies on whether whey protein or casein can modulate inflammation and immune response in individuals with impaired metabolic control. A recent study in obese non-diabetic individuals given a high fat mixed meal and whey protein isolate revealed the CCL5 response, an indicator of immune activation, in the 4 h postprandial period [86]. In addition, the MCP-1 response was higher after the whey protein isolate compared to other proteins [86]. Another recent study in patients undergoing surgery reported that a whey protein and CHO drink decreased the postoperative acute phase response and insulin resistance [87]. Conversely, in a group of overweight postmenopausal women, a 45 g whey protein isolate or sodium caseinate bolus did not decrease postprandial plasma inflammatory markers, IL-6, TNF-α or acute phase C-reactive protein (CRP) over a 6 hour period compared to glucose ingestion [81]. Similarly, in a longer term study in overweight and obese individuals, whey protein or casein supplementation for 12 weeks was reported not to influence plasma inflammatory markers [77]. However, in patients with chronic obstructive disease (COPD) undergoing a low-intensity exercise therapy, whey peptide was found to decrease circulating IL-6, IL-8, TNFα and hsCRP, accompanied by an increased exercise tolerance [88]. While *in-vitro* evidence indicates that whey protein and casein have immunomodulatory effects, more *in-vivo* studies are required to assess whether these proteins can modulate the immune response in individuals with impaired metabolic health and low-grade inflammation.

### Milk proteins and appetite control

In addition to the direct effects of dairy protein on metabolic health, milk proteins may indirectly drive improvements in metabolic control through the enhancement of the control of appetite and/or other mechanisms which aid changes in both body weight and composition. High-protein foods have long been shown to have favourable effects on satiety [89-95], and whilst not all studies show suppression of food intake [96-99], altering the diet in favour of a higher protein component may well have a role to play in weight control [100-105]. The significant advantage of a high dairy diet for appetite suppression and weight loss is the anabolic effect of dairy BCAAs on lean body mass. BCAAs enhance muscle protein synthesis and skeletal muscle mass, and may protect against loss of lean mass during periods of weight loss [106]. As the largest organ group in the body and a highly metabolically active tissue, protection of skeletal mass may in turn contribute to an improvement in whole body metabolic health.

Whether different protein types have different effects on food intake however is not as yet well demonstrated, although there are some studies which have shown greater satiety for some dairy proteins relative to soy protein [107,108] or other non-dairy proteins [109]. Interestingly skimmed milk containing both whey protein and casein has been shown to decrease intake more than either protein alone in a study of isoenergetic preloads [110], whilst in another study whey protein in turn has been shown to suppress intake more than casein [38]. Various whey protein-derived fractions may also have differential effects on satiety, including GMP which has long been purported to suppress food intake. Supportive evidence however is lacking [106,107]. In an isoenergetic study of whey protein, whey protein plus GMP, whey protein-derived alpha-lactalbumin (α-lac), casein and non-dairy soy protein, it was α-lac that was shown to suppress intake relative to the other fractions [111], whilst in a study comparing various forms of GMP no differential effects on intake were reported [112]. In a recent study of overweight women from our laboratory we also found no differential effect of GMP on energy intake [113] when we gave matched beverages containing 25 g of whey protein concentrate, GMP, beta-lactoglobulin (β-lac) and colostrum-derived whey protein, although greater fullness was induced by β-lac [113].

In one of the early studies, protein-induced appetite suppression was attributed to both an altered rate of gastric emptying and an increase in postprandial serum AA concentrations following whey protein consumption [38]. Differential effects have also been attributed to threshold concentrations of total serum AAs in more recent studies of whey protein-induced hunger suppression when whey and casein were given in a mixed preload meal [107]. Interestingly in this study a lower dose (15 g, 10 en% protein) had a greater effect than a higher dose (38 g, 25 en% protein) [107]. Despite some differential effects of whey protein fractions in our recent study [113], we were unable to find a relationship between circulating levels of total AAs and measures of either hunger, satiety or food intake. It is possible that tryptophan may be the most important of the AAs for appetite suppression since 5-hydroxytryptophan (5HT, serotonin) is an established appetite-modulating neurotransmitter [107], but this remains as yet unsubstantiated. Other postulated mechanisms include regulation of satiety by the gastrointestinal (GI) peptides such as glucagon like peptide-1 (GLP-1), CCK and peptide YY (PYY) [114-116]. Circulating levels of these peptides clearly are altered following a meal where they have a role in digestion, absorption and the metabolic fate of ingested nutrients, but there remains questions around their role in the control of hunger, satiety and eating behavior [97,117]. Our review of the literature and that of others [118] leads to the conclusion that the role played by these peptides in the control of food intake is poorly demonstrated. De Graaf et al., [118] observed that peptide concentrations induced through exogenous (pharmaceutical) administration are several-fold greater than those which occur when a meal is consumed [118], Whilst GLP-1 and PYY have clear anorectic effects at high pharmacological levels, following a meal blood concentrations remain relatively low and neither is likely to contribute significantly to the satiating effect of protein or other macronutrients [118]. Circulating CCK levels following a high protein meal are closer to those achieved following exogenous infusion, yet the evidence that CCK is a critical driver in human satiety also remains elusive.

Unsurprisingly, the postprandial effects of dairy protein on GI hormones and energy intake are mixed, with changes in peptide concentrations rarely driving predictable changes in energy intake [118]. For example, in a study of lean and obese men, 50 g of whey protein resulted in a prolonged postprandial suppression of ghrelin, increased GLP-1 and CCK, and as hypothesized, decreased energy intake of ~10% [95]. Conversely, a study in obese men reported that 50 g whey protein resulted in a prolonged suppression of ghrelin, elevation of GLP-1 and CCK [97], but no detectable changes in energy intake [97]. Hence, changes in gut satiety peptides in response to pre-meal protein beverages or after a meal do not reliably translate into changes in subjective appetite and satiety ratings or energy intake.

### Milk proteins and body weight

Weight-loss diets are widely undertaken to lose excess abdominal fat, which has implications for metabolic health since visceral adipose tissue produces inflammatory cytokines, which can inhibit insulin action in skeletal muscle. Recent evidence has highlighted the role that dietary protein may play in weight control [105], with a large scale European Union study, DIOGENES, reporting that long-term weight loss maintenance was obtained most effectively in overweight individuals allowed to eat a high-protein, low glycaemic index (GI) diet [105]. There have been several long-term studies of the effect of dairy protein on body composition both in the presence or absence of a weight-loss or weight-loss maintenance diet, which are reviewed below.

### Ad libitum diets for weight-loss

In a randomized controlled trial, supplementation with ~56 g/day whey protein concentrate for 6 months without any dietary advice resulted in significantly lower body weight, fat mass and waist circumference in overweight and obese individuals compared to an isoenergetic CHO control [108]. In a second, shorter trial of overweight and obese individuals with pre-existing metabolic risk however, 54 g/day of whey protein isolate for 3 months failed to cause weight loss, but notably metabolic health improved through decreased fasting blood lipids and insulin levels [59].

### Energy restricted diets for weight-loss

Whilst an energy-restricted diet provides an effective way to decrease adipose tissue mass and abdominal obesity, energy intake below basal metabolic requirements also leads to skeletal muscle loss. Weight-regain after an energy restricted diet often occurs, in part due to the decrease in basal metabolic rate (BMR) resulting from loss of metabolically active skeletal muscle mass. In middle-aged and older obese participants existing muscle sarcopenia may further exacerbate this weight-regain [6]. The effectiveness of whey protein in conjunction with dietary advice to improve adiposity and lean body mass in addition to body weight *per se* has been assessed in several studies. Whey protein is well established as a driver of skeletal muscle anabolism, with its constituent BCAAs such as leucine driving greater protein synthesis [119,120]. As such, a high dairy protein diet may enhance metabolic health through maintenance of lean body mass during weight loss. In a randomized trial of obese individuals on an energy restricted diet, a milk protein based supplement lead to higher fat loss and preservation of lean mass, as well as greater overall weight-loss [121]. Another study in obese men also reported that consumption of a high protein meal replacement containing whey protein, soy protein and AAs resulted in greater adipose loss over 12 weeks compared to a standard protein meal replacement plan [122]. A recent study of overweight elderly has also shown that supplementation with whey protein plus EAAs during an energy restricted diet promoted skeletal muscle protein synthesis compared with standard meal replacements, and in turn promoted greater adipose loss [123]. Unexpectedly in this study however there was no increase in skeletal muscle mass during EAA supplementation. Conversely, a shorter-term study of whey protein supplementation during 8 weeks of weight-reduction followed by 12 weeks weight-maintenance did not alter body weight or composition compared to a CHO control, although the small sample size of only 18 participants greatly limited the power to detect changes in fat or lean tissue mass and raises questions on the findings of this trial [98].

Whether whey protein or casein may be more effective for adipose loss and/or skeletal mass sparing during energy restriction is not known. One recent study in obese individuals on a 6 week energy restricted, weight loss diet with ~60 g/day casein or whey protein supplementation reported no differences in fat or lean mass changes between the groups [124]. Casein supplementation did however result in greater inhibition of protein degradation despite lower protein synthesis, and an overall greater positive protein balance. Possibly the duration of the study was too short for effects on body composition changes to be detected [124]. In a longer study, in obese individuals with increased metabolic risk, both whey protein and casein supplementation were more effective for maintenance of body weight and adipose-loss over 12 weeks compared to CHO supplementation, but no differences were found between the whey protein or casein supplemented groups [125]. Taken together, an energy restricted diet combined with whey protein or casein supplementation may help to enhance body weight and adipose loss whilst maintaining skeletal muscle mass essential for good metabolic control. There is however no consensus in support of differential effects of whey protein versus casein for weight-loss or improved body composition.

### Milk proteins and maintenance of metabolically active muscle

Skeletal muscle mass is determined by net balance between the synthesis of new proteins and degradation of existing proteins. In addition, storage of CHOs in the form of glycogen and lipids in the form of intramyocellular TGs contribute to minor fluctuations in skeletal muscle mass. Daily turnover of skeletal muscle protein in healthy recreationally active humans is around 1-2% day [126]. Protein synthesis is increased postprandially several times a day and decreased during fasting. The postprandial response appears to be gradually blunted during aging however, and this lower rate of muscle protein synthesis may be a key factor in aging sarcopenia [127]. Exercise is also important, and there is evidence that skeletal muscle turnover in young, sedentary individuals may be lower than active, elderly individuals [128]. Regular dairy protein supplementation in individuals with impaired metabolic control exacerbated by poor muscle mass, often a result of physical inactivity and/or ageing, has been postulated to prevent further loss of lean mass and promote skeletal muscle accretion and enhanced function.

### Enhanced muscle mass and function

Milk proteins provide a potent anabolic stimulus due to their AA composition and insulinotropic effects, although whether whey protein or casein have greater differential effects on muscle mass and/or function is not yet well understood. An early study in the mid 1990s found that postprandial whole body protein synthesis was increased 68% after ingestion of whey protein but only 31% after ingestion of casein [40]. However, casein but not whey inhibited whole body protein breakdown and so despite the more rapid appearance of whey-derived AAs, net protein balance was higher following casein ingestion [40]. Similar observations have been made in later studies where the slow digestion of casein or milk protein concentrate resulted in a more sustained appearance of AAs and protein accretion, despite lower peak levels of BCAAs [41,129]. There is also temporal variation in protein synthesis rates, with a recent study using intrinsically labeled whey protein and casein co-ingested as milk showing that AA absorption and retention across a lower limb were similar for both whey protein and casein-derived AAs for the first 2 hours, but were higher in casein-derived AAs after >3 hours [130]. Conversely in a study of older men [37], 20 g of whey protein resulted in greater postprandial muscle accretion over 6 hours compared to either casein or casein hydrolysate, despite similar peak rates of serum AA appearance between whey protein and hydrolysed casein. This was attributed to the higher leucine content of whey protein [37], which in combination with its insulinotropic effects may be important to overcome insulin resistance in individuals with poor metabolic control. Whether post-prandially induced increases in protein synthesis by milk proteins is sufficient to result in net skeletal mass accretion over time without exercise is yet to be established. Resistance exercise alone provides an anabolic stimulus, therefore ingestion of milk protein is likely to be more advantageous in combination with exercise interventions to improve metabolic health.

### Synergies with exercise

Progressive exercise training causes multiple physiological adaptations in skeletal muscle, which underpin increased insulin sensitivity, metabolic control and functional changes. These physiological adaptations require synthesis of new proteins and breakdown of existing proteins. After acute resistance or endurance type exercise, protein synthesis and protein breakdown are transiently increased dependent upon both the work load and intensity [126]. Milk protein ingestion in combination with resistance exercise training may result in greater skeletal muscle hypertrophy, and in turn increased insulin sensitivity, metabolic control and BMR. On the same premise milk protein ingestion may be postulated to accelerate the metabolic adaptation to either resistance or endurance type exercise training. The main evidence for this is based on acute increases in whole body or muscle protein synthesis rates after exercise following ingestion of milk proteins. For example, after resistance exercise, acute ingestion of 20 g of both whey protein and casein resulted in an increase in net muscle protein balance [131]. Direct assessment of myofibrillar protein synthesis rate 1-6 h post exercise revealed similar increases after 20 g dose of whey protein or casein, although as expected whey produced a marked increase in the early period whereas casein produced a more moderate but prolonged increase [44]. Acute, early phase, post exercise muscle protein synthesis was also higher following whey protein hydrolysate compared to casein [132]. By far the majority of studies of milk protein supplementation on skeletal muscle synthesis have been conducted in young healthy individuals or the elderly. Protein synthesis may be blunted with insulin resistance, therefore whether milk proteins can enhance post-exercise muscle protein synthesis and also longer term exercise-induced metabolic adaptations in obesity, metabolic syndrome or T2DM is of great interest.

Two recent studies in overweight individuals, where chronic exercise training was combined with milk protein supplementation, provide little consensus for increases in muscle mass and metabolic adaptation. In a 6 week randomized controlled trial in overweight young men, 3 × 30 g/day whey protein isolate combined with resistance exercise did not result in greater improvement in metabolic risk compared to a placebo of starch [133]. The test supplements were ingested immediately after exercise, and also with lunch and dinner each day. The duration of the intervention was relatively short, given that metabolic adaptations to training, such as increased muscle mass and insulin sensitivity, may take over 12 weeks to occur [134]. In a longer-term 6 month study of weight loss in overweight, older women, 2 × 25 g/day whey protein isolate in conjunction with an energy restricted diet and exercise training resulted in both a trend towards greater weight loss, and MRI-assessed adipose loss and gain in leg muscle mass [135]. Perhaps unexpectedly there is as yet limited evidence even in young healthy participants and bodybuilders that protein supplementation in combination with resistance exercise training does lead to greater gains in skeletal muscle mass [136-138], despite the growing literature supporting effects on protein synthesis, degradation and balance. In a study of elderly men undertaking a 12 week progressive resistance type exercise program, the addition of a protein supplement, perhaps surprisingly, did not further increase skeletal muscle hypertrophy and enhance lean body mass [139]. Habitual protein intake may be a factor which influences the potential benefits of dairy supplementation in combination with exercise.

## Conclusions

Epidemiological evidence shows dairy consumption to be associated with a decreased prevalence of metabolic related disorders, supported by experimental studies showing milk protein decreases the prevalence of individual metabolic risk factors such as hypertension, dyslipidaemia, and mild hyperglycaemia. As well as via direct mechanisms, there is also evidence that milk protein may indirectly improve metabolic health by promoting changes in body composition in favour of increased lean body mass and decreased adiposity, particularly during energy-restricted weight loss. BCAAs, present in high levels in milk protein, enhance muscle protein synthesis, lean body mass and skeletal muscle metabolic function. ‘Sarcobesity’, where the adverse metabolic profile of overweight and obesity is superimposed onto a declining, inadequate and poorly functional skeletal muscle mass, is a rapidly growing health issue, and lifestyle changes which include an energy restricted diet and increased activity can lead to significant improvements in metabolic health. Whey protein supplementation has positive effects on postprandial and post-exercise glucose, lipid and protein metabolism and may prevent declining metabolic health if used in conjunction with lifestyle changes. Large randomized milk protein supplementation trials combined with lifestyle changes are still required however to generate robust evidence supporting the use of milk protein products to improve or manage metabolic health.

## Competing interests

SDP holds the Fonterra Chair in Human Nutrition at the University of Auckland. RAM receives financial support from the New Zealand Primary Growth Partnership (PGP) program, funded by Fonterra Co-operative group and the NZ Ministry for Primary Industries (MPI).

## Authors’ contributions

RAM and SDP wrote the manuscript and are responsible for all content. Both authors read and approved the final manuscript.
